# Nanoemulsion and Nanogel Containing *Eucalyptus globulus* Essential Oil; Larvicidal Activity and Antibacterial Properties

**DOI:** 10.1155/2022/1616149

**Published:** 2022-08-31

**Authors:** Hiva Alipanah, Abbas Abdollahi, Samira Firooziyan, Elham Zarenezhad, Mojtaba Jafari, Mahmoud Osanloo

**Affiliations:** ^1^Department of Physiology, School of Medicine, Fasa University of Medical Sciences, Fasa, Iran; ^2^Department of Microbiology, School of Medicine, Fasa University of Medical Sciences, Fasa, Iran; ^3^Urmia Health Center, Disease Control Unit, Urmia University of Medical Sciences, Urmia, Iran; ^4^Noncommunicable Disease Research Center, Fasa University of Medical Sciences, Fasa, Iran; ^5^Student Research Center Committee, Fasa University of Medical Sciences, Fasa, Iran; ^6^Department of Medical Nanotechnology, School of Advanced Technologies in Medicine, Fasa University of Medical Sciences, Fasa, Iran

## Abstract

*Eucalyptus globulus* essential oil (EGEO) possesses many biological effects such as antibacterial, antifungal, and insecticide properties. In the current study, the chemical composition of EGEO was first investigated using GC-MS analysis. Then, a nanoemulsion and nanogel containing EGEO (EGEO-nanoemulsion and EGEO-nanogel) were prepared. After that, the successful loading of EGEO was confirmed using ATR-FTIR analysis. EGEO-nanoemulsion and EGEO-nanogel with LC_50_ values of 27 and 32 *μ*g/mL showed promising efficacies against *Anopheles stephensi* larvae. Besides, the efficacy of EGEO-nanogel (IC_50_ 187 *μ*g/mL) was significantly more potent than EGEO-nanoemulsion (IC_50_ 3732 *μ*g/mL) against *Staphylococcus aureus.* However, no significant difference was observed in the efficacy of EGEO-nanoemulsion and EGEO-nanogel against *Pseudomonas aeruginosa.* Natural components, straightforward preparation, and proper efficacy are some of the advantages of EGEO-nanogel; it could be considered for further consideration against other pathogens and mosquito larvae.

## 1. Background

*Eucalyptus globulus* (Family *Myrtaceae*) is a fast-growing evergreen and magnificent tree cultivated worldwide [1]. Its extracts have been traditionally used for tuberculosis, bacterial diarrhea, respiratory infections, joint pain, and similar cases [2, 3]. Moreover, the antioxidant activity of *E. globulus* and other *Eucalyptus* species, including *E. citriodora, E. camaldulensis, E. microtheca,* and *E. sargentii,* has been reported [4, 5]. Besides, the antibacterial effects of its essential oil (EGEO) on multidrug-resistant microorganisms have been confirmed [6–8]. For instance, EGEO affects *Pseudomonas aeruginosa, Escherichia coli,* and *Staphylococcus aureus* [6, 9]. Moreover, *S. aureus* and *P. aeruginosa* are opportunistic pathogens that cause acute and chronic hospital-acquired and respiratory tract infections [10, 11].

About 241 million malaria cases were identified worldwide in 2020, of which 627,000 people lost their lives [12]. *Anopheles stephensi* mosquitoes, as a domestic species, are one of the most important malaria vectors [13]. Insecticides have maintained a proven and effective tool for malaria control [13]. However, physiological and behavioral resistance in mosquito vectors such as *A. stephensi* is now a great threat to human health [14]. Therefore, using nonchemical or biopesticides (such as EO-based pesticides) to protect the environment and prevent mosquito resistance is a promising way to combat malaria vectors [15]. For example, the larvicidal activity of *E. camaldulensis* EO with an LC_50_ value of 397.75 ppm against *A. stephensi* has been reported [16]. Furthermore, experimental laboratory data showed that *E. tereticornis* EO inhibited the pupal and adult activity of *A. stephensi* and showed nearly 100% mortality rate at the highest dose (160 ppm) [17].

Nanoemulsions with small droplets, optical transparency, and long-term physical stability (without coagulation, deposition, or biphasic) are easily absorbed by microorganisms. Therefore, their bioavailability is high and their efficiency increased compared to the nonnano state [18–20]. Despite the mentioned advantages, topical usage of nanoemulsions is challenging due to their low viscosity. In recent years, this challenge has been met by gelling the nanoemulsions, while the benefits of the nanoemulsion still exist [21, 22].

In this study, the chemical composition of EGEO was first investigated. After that, nanoemulsion and nanogel containing EGEO (EGEO-nanoemulsion and EGEO-nanogel) were prepared. Finally, their antibacterial and larvicidal activities against *P. aeruginosa, S. aureus,* and *A. stephensi* were investigated.

## 2. Methods

### 2.1. Materials

Standard species of *P. aeruginosa* and *S. aureus* (ATCC 27853 and 25923) were obtained from the Pasteur Institute of Iran. *A. stephensi* late 3^rd^ or early 4^th^ instar larvae were provided by Urmia University of Medical Sciences (Iran). Mosquitos were reared and kept at 29 ± 2°C with 70 ± 5% humidity. *E. globulus* EO bark' EO was purchased from Tabib Daru pharmaceutical company (Iran) (TaDa 1729). Carboxymethylcellulose (CMC: C4888) was bought from Sigma–Aldrich (USA). Mueller Hinton agar (103872), Mueller Hinton broth (110293), and Tween 20 (817072) were bought from Merck Chemicals (Germany).

### 2.2. GC-MS Procedure

EO constituents were analyzed by a GC-MS system (Santa Clara, CA, USA) using a 6890 gas chromatography and a 5975 mass selective detector (Agilent Technologies, USA). The column was an HP-5MS silica-fused (length, 30 m; internal diameter, 0.25 mm; film thickness, 0.25 mm; stationary phase, 5% phenyl, and 95% methyl polysiloxane). The column temperature profile was as follows: 40°C (fixed for 1 min), then raised to 250°C at the rate of 3°C min^−1^ and held for 60 minutes. The temperatures of the injection port and detector were set at 250 and 230°C, respectively. Helium (99.999%) was used as a carrier gas. The septum purge and column flow rate were fixed at 6 mL/min and 1 mL/min, respectively. Mass spectra were taken at full scan mode at 50–550 m/z with 70 eV ionization energy. Van den Dool and Kratz formula was applied to calculate the retention indices of n-alkanes [23]. The identification of the EO components was approached by combining the temperature programmed retention indices and mass spectra from ADAMS and the NIST 17 mass spectra library. Besides, relative abundances were obtained by peak area normalization [24, 25].

### 2.3. Preparation and Characterizations of EGEO-Nanoemulsion and EGEO-Nanogel

The nanoemulsions were prepared using the spontaneous method as reported in our previous study [26]. The EGEO (2% v/v) was first mixed (2000 rpm, 5 minutes) with different amounts of Tween 20 (2–6% v/v) at room temperature. Distilled water was then added drop wisely until reached 5000 *μ*L (final volume) and stirred for 40 minutes (room temperature). Finally, prepared nanoemulsions were subjected to a DLS-type apparatus for size analysis. EGEO-nanoemulsion with optimal values (droplet size <200 nm and droplet size distribution (SPAN) <1) [27] was selected for further investigation, including preparation of EGEO-nanogel, antibacterial tests, and antilarval bioassays.

The optimum EGEO-nanoemulsion was gelified as follows. Then, CMC (3.5% w/v) was first added to the EGEO-nanoemulsion and the mixture was then stirred at 2000 rpm for 4 h at room temperature to complete the gelation process. The viscosity of EGEO-nanogel at shear rates of 0.1–10 1/s was analyzed by the rheometer apparatus (Anton Paar, model MCR-302, Austria) under atmospheric pressure at 25°C. Moreover, nanoemulsion (-oil) and nanogel (-oil) were also prepared using the same method, only without *E. globulus* EO.

ATR-FTIR analysis was used for the investigation of the loading of EGEO in EGEO-nanoemulsion and EGEO-nanogel. Spectra of EGEO, nanoemulsion (-oil), nanogel (-oil), EGEO-nanoemulsion, and EGEO-nanogel were recorded in the wavenumber range of 400–4000 cm^−1^ [28]. The samples were subjected to the device (Tensor II model, Bruker Co, Germany) without any sample processing.

Furthermore, EGEO-nanoemulsion stability was checked using three tests. First, EGEO-nanoemulsion was 30 min centrifuged (14,000 g) at −4, +4, and +25°C, 30 min. Second, EGEO-nanoemulsion was subjected to freeze-thaw cycle tests; samples were placed for 48 h at −20°C (freezer) and room temperature for six successive periods. Third, EGEO-nanoemulsion was subjected to heating-cooling cycle tests; samples were placed for 48 h at +45°C (Bain–Marie) and room temperature for six successive periods. After each test, samples were visually checked for sedimentation, creaming, or biphasic. Moreover, EGEO-nanogel samples were stored at 4 and 26°C for six months and then were visually checked for sedimentation, creaming, or biphasic [29].

### 2.4. Larvicidal Bioassays

Larvicidal bioassays of EGEO-nanoemulsion and EGEO-nanogel were carried out according to the WHO recommended process [30]. Glass beakers containing 200 mL no-chlorine water and 25 larvae of *A. stephensi* were first prepared. The larvicidal effects of the EGEO-nanoemulsion and EGEO-nanogel were then investigated at 12.5, 25, 50, 100, and 150 *μ*g/mL. After 24 h exposure, larval mortality rates were recorded. The control and blank groups were treated with ethanol and nanoemulsion (-oil) and nanogel (-oil).

### 2.5. Antibacterial Tests

The antibacterial properties of EGEO-nanoemulsion and EGEO-nanogel were investigated using the ATCC100 method [22]. First, aliquots of samples were sequentially diluted in 5 cm plate plates containing each bacterial suspension (2 × 10^5^ CFU/mL in the Mueller Hinton broth) to adjust final concentrations of 1250, 2500, and 5000 *μ*g/mL. Then, treated plates were incubated at 37°C for 24 h and 10 *μ*L-microbial suspensions were cultured on the Mueller Hinton agar plates and incubated for 24 h. The number of bacterial colonies forming units (CFU) was counted, and growth (%) was calculated by the following formula: (CFU sample/CFU control) × 100.

Furthermore, MIC (Minimum Inhibitory Concentration) and MBC (Minimum Bactericidal Concentration) values are determined in semiquantitative assays such as disc or well diffusion methods. This study used ATTCC100 as a quantitative method; it investigated antibacterial effects, % bacterial growth, or % bacterial growth inhibitory, at different concentrations. The IC_50_ value is measured this way, as calculated in this study.

### 2.6. Statistical Analyses

Three replicates were carried out for all tests and final values were reported as mean ± SD. The final values for all samples were compared with SPSS software using one-way ANOVA with a confidence interval of 95%. Turkey's test evaluated statistical differences between groups. Besides, IC_50_ (half-maximal inhibitory concentration) and LC_50_ (Lethal concentration 50) values were calculated using CalcuSyn software (Free version, BIOSOFT, UK).

## 3. Results

### 3.1. Ingredients of EGEO

Eleven identified compounds in EGEO using GC-MS analysis are listed in Table 1. Five major compounds are 1,8-cineole (49.53%), *α*-pinene (15.33%), *trans*-pinocarveol (4.32%), aromadendrene (3.95%), and globulol (3.69%).

### 3.2. Prepared EGEO-Nanoemulsion and EGEO-Nanogel

**Figure 1:**
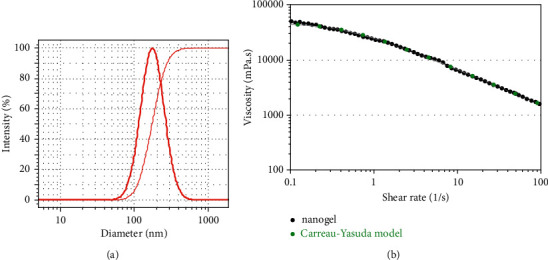
(a) DLS analysis of EGEO-nanoemulsions with a droplet size of 176 ± 8 nm and (b) viscosity of the EGEO-nanogel.

The ingredients of five prepared EGEO-nanoemulsions and their size analysis are listed in Table 2. Sample No. 5 with droplet size (176 ± 8) and SPAN (0.97) showed the best size characteristics; its DLS profile is shown in Figure 1(a). Besides, the viscosity of the EGEO-nanogel at examined shear rates (0.01–100 1/s) is entirely consistent with the Carreau–Yasuda regression (Figure 1(b)). This well-known empirical equation has been used for nonNewtonian fluids such as biopolymer and polymeric solutions, emulsions, and protein solutions [31, 32].

Moreover, no creaming, sedimentation, and phase separation were observed in EGEO-nanoemulsions after all stability tests, including centrifugation (at −4, +4, +25°C), freeze-thaw cycles, and heating-cooling cycles. Besides, EGEO-nanogel did not show any biphasic or phase separation after six months of storage at 4 and 26°C. Therefore, the stability of EGEO-nanoemulsion and EGEO-nanogel was confirmed.

### 3.3. ATR-FTIR Spectra

**Figure 2:**
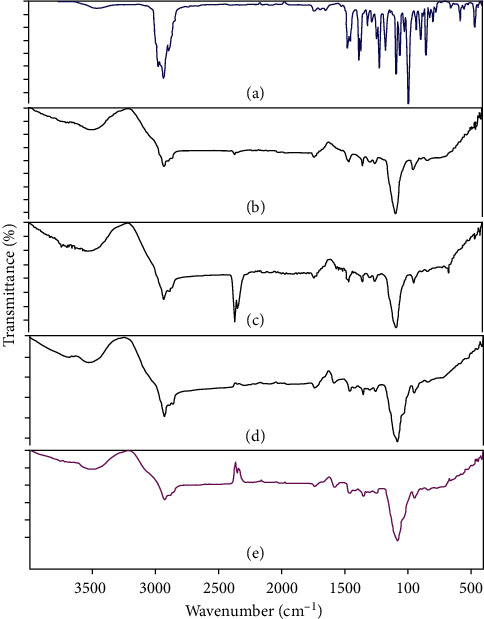
ATR-FTIR spectra of A: *E. globulus* EO (EGEO), B: nanoemulsion (-oil), C: EGEO-nanoemulsion, D: nanogel (-oil), and E: EGEO-nanogel.

The ATR-FTIR spectra of EGEO, nanoemulsion (-oil), EGEO-nanoemulsion, nanogel (-oil), and EGEO-nanogel are presented in Figure 2. In the ATR-FTIR spectrum of EGEO, broadband at about 3450 cm^−1^ can be related to the OH group. The characteristic bands at 2880, 2921, and 2966 cm^−1^ are attributed to CH stretching vibration, and the bands at about 1682 and 1644 cm^−1^ can correspond to C=C stretching vibration in olefin. The strong peak at 1446 cm^−1^ is allocated to symmetrical bending in the plane of C-H bonds and the strong band at 1375 cm^−1^ can be related to CH_3_ deformation. The bands at 1079 and 1214 cm^−1^ corresponded to symmetric and asymmetric stretching of the C-O-C group. A sharp and strong band at 984 cm^−1^ can be attributed to symmetrical bending out of the CH_2_ plane.

In ATR-FTIR spectra of nanoemulsion (-oil), the broad peak at about 3200–3600 cm^−1^ is attributed to OH stretching vibration due to hydrogen bonding between tween and water. The band at 2923 cm^−1^ corresponds to the C-H stretching. The characteristic peak at 1732 cm^−1^ is related to C=O stretching due to the carbonyl group in tween 20. Besides, the characteristic absorption at around 1457 cm^−1^ exhibited CH_2_ bending. Finally, the strong and characteristic band at 1087 cm^−1^ is attributed to C-O stretching.

The ATR-FTIR spectrum of EGEO-nanoemulsion displayed the broad and characteristic band at around 3200–3700 cm^−1^ can be related to OH groups in tween 20, H_2_O, and EO that lead to hydrophilic interaction. The peak at about 2923 cm^−1^ is related to C-H stretching due to EO and tween 20. The bands at 2341 and 2359 can be related to CO_2_ and the band at 1733 cm^−1^ is attributed to the carbonyl group in Tween 20. The band at 1457 cm^−1^ can be related to CH_3_ bending in EO and Tween 20. Totally between 400 and 4000 cm^−1^, the intensities of the EGEO-nanoemulsion were higher than those in nanoemulsion (-oil), which is a sign of successful loading of EGEO in EGEO-nanoemulsion.

In the ATR-FTIR nanogel (-oil) spectrum, broadband in the region 3200–3600 cm^−1^ can be related to OH due to hydrogen bonding. The peaks at about 2923 and 2855 cm^−1^ are related to C-H stretching due to CMC. The characteristic band at 1734 cm^−1^ related to C=O stretching is due to the carbonyl group in Tween 20 and CMC. The characteristic band at about 1577 cm^−1^ is attributed to the carboxylate group's asymmetric stretching. Besides, the peak at about 1417 cm^−1^ corresponds to CMC's symmetric stretching of the carboxyl group. Finally, the strong band at about 1081 cm^−1^ can be attributed to C-O stretching vibration.

In the ATR-FTIR spectrum of the EGEO-nanogel, a broadband in the region 3200–3500 cm^−1^ corresponded to OH due to hydrogen bonding in CMC, Tween 20, and the EO. The band at around 2923 cm^−1^ is related to C-H stretching due to the EO, Tween 20, and CMC. The band at 1734 cm^−1^ showed C=O stretching that exhibited the carbonyl group in the EO, Tween 20, and CMC. The sharp and strong band at 1080 cm^−1^ corresponded to C-O stretching. In the presence of CMC, the carboxylate band at 1579 cm^−1^ demonstrated intramolecular H-bonding between Tween 20 and CMC. The physical interaction between the surface –OH of the Tween 20 and the –OH groups of the CMC molecule led to the consumption of a small amount of –OH groups. The appearance of the other bands in EGEO and nanogel (-oil) confirmed the successful loading of EGEO in the prepared EGEO-nanogel

### 3.4. Antibacterial Effects

**Figure 3:**
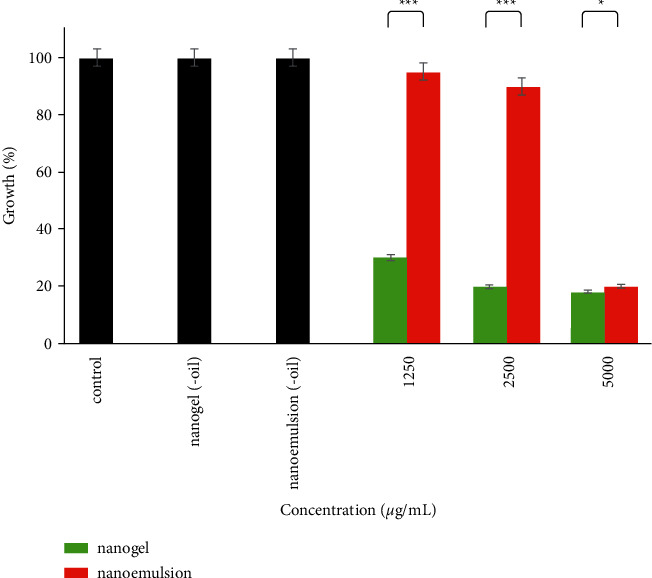
Antibacterial activities of EGEO-nanogel and EGEO-nanoemulsion against *S. aureus*. ^*∗*^: *P* < 0.05, ^*∗∗∗*^: *P* < 0.001.

The antibacterial activities of EGEO-nanoemulsion and EGEO-nanogel against *S. aureus* are illustrated in Figure 3. As details show, the efficacy of EGEO-nanogel was more potent than EGEO-nanoemulsion at all examined concentrations, including 1250 *μ*g/mL (*P* < 0.001), 2500 *μ*g/mL (*P* < 0.001), and 5000 *μ*g/mL (*P*=0.012). Interestingly, about 80% of bacterial growth was reduced after treatment with 5000 *μ*g/mL EGEO-nanogel and EGEO-nanoemulsion. Besides, nanoemulsion (-oil) and nanogel (-oil) did not affect the growth of bacteria. Furthermore, EGEO-nanogel with IC_50_ 187 *μ*g/mL was significantly more potent (*P* < 0.05) than EGEO-nanoemulsion (Table 3).

**Figure 4:**
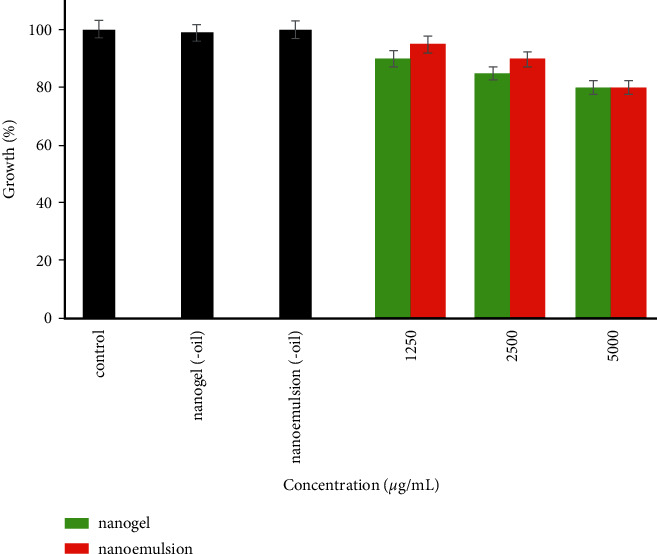
Antibacterial activity of EGEO-nanogel and EGEO-nanoemulsion against *P. aeruginosa*.

The antibacterial activities of EGEO-nanoemulsion and EGEO-nanogel *P. aeruginosa* are depicted in Figure 4. There is no significant difference observed between the efficacy of EGEO-nanogel and EGEO-nanoemulsion. Besides, their efficacy on *P. aeruginosa* was less than *S. aureus*; only 20% of bacterial growth was reduced after treatment with 5000 *μ*g/mL EGEO-nanogel and EGEO-nanoemulsion. Besides, the nanoemulsion (-oil) and nanogel (-oil) did not affect the growth of *P. aeruginosa*. As efficacies of EGEO-nanogel and EGEO-nanoemulsion at the highest concentration (5000 *μ*g/mL) were less than 50%, their IC_50_ values were not determined (Table 3).

### 3.5. Larvicidal Effects

**Figure 5:**
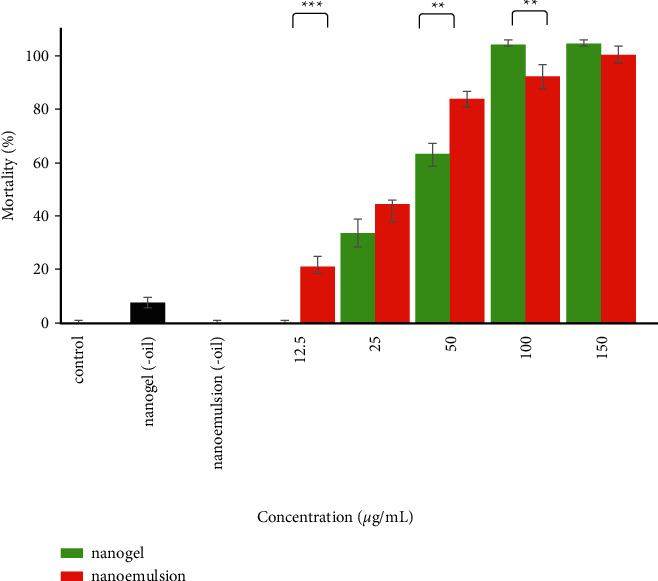
The larvicidal activities of EGEO-nanogel and EGEO-nanoemulsion against *A. stephensi*. ^*∗∗*^: *P* < 0.01, ^*∗∗∗*^: *P* < 0.001.

The larvicidal activities of EGEO-nanoemulsion and EGEO-nanogel against *A. stephensi* are demonstrated in Figure 5. A dose-response effect was observed in the mortality rate of *A. stephensi.* The efficacy of EGEO-nanoemulsion was significantly more potent than EGEO-nanogel at concentrations of 12.5 (*P* < 0.001) and 50 *μ*g/mL (*P*=0.002), however, the efficacy of EGEO-nanogel at a concentration of 100 *μ*g/mL was significantly more potent than EGEO-nanoemulsion (*P*=0.007). Interestingly, perfect larval mortality was observed at two concentrations of EGEO-nanogel (100 and 150 *μ*g/mL). However, as summarized in Table 3, the LC_50_ values of EGEO-nanogel and EGEO-nanoemulsion were not significantly different (32 and 27 *μ*g/mL). Moreover, nanoemulsion (-oil) did not affect larvae and nanogel (-oil) with 6% mortality had a negligible effect on larvae.

## 4. Discussion

Microbial infections and mosquito-borne diseases are still major public health challenges. *S. aureus* is one of the most important pathogens of food-borne diseases and community-associated infections worldwide. It is resistant to harsh environmental conditions and is highly stable in different temperatures (7 to 48.5°C), pH (4.2 to 9.3), and 15% NaCl concentrations [33, 34]. Besides, *P. aeruginosa* plays a major role in increased morbidity and mortality rates in respiratory diseases like cystic fibrosis. Moreover, antibiotic resistance in *P. aeruginosa* due to low permeability in the outer membrane has created many therapeutic challenges [35].

Furthermore, excessive use of insecticides has led to resistance in mosquito populations and environmental pollution [36, 37]. The development of natural medicine and insecticides is thus crucial. EOs with a wide range of biological activities such as antibacterial, antioxidant, anticancer, and antilarval effects are a great source for this purpose [38, 39]. However, their efficacy and stability should be improved for practical application. Nowadays, nanostructured-loaded EOs are considered a promising approach [40]. Nanostructures can also increase their solubility and improve stability and effectiveness [41, 42].

EGEO in the present study was used as a natural antibacterial and larvicide. Therefore, its ingredients were first identified using GC-MS analysis. 1,8-cineole was the most abundant component, followed by *α*-pinene (15.33%), *trans*-pinocarveol (4.32%), aromadendrene (3.95%), and globulol (3.69%). These findings were consistent with the literature that 1,8-cineole and *α*-pinene were introduced as two major components of EGEO or other *Eucalyptus* spp. EOs [43, 44]. For example, 1,8-cineole was the major ingredient in eight *Eucalyptus* species' essential oils from Tunisia [44]. Differences in the levels of major compounds may be due to genetic effects or harvester place.

Furthermore, some studies have been published on the antibacterial effects of eucalyptus extract on *E. coli*, *S. aureus,* and *P. fluorescens* [45–47]. Besides, it has been confirmed that inhaling eucalyptus extracts benefit non/infectious respiratory disorders, such as bronchitis, asthma, and chronic obstructive pulmonary disease [48, 49]. Moreover, EGEO is an immune stimulant with antiinflammatory, antioxidant, and analgesic effects [48, 49]. Some reports on its nanoformulated states were also reported. For example, its nanoemulsion showed wound healing potential without skin irritation in Wistar rats [50]. Moreover, larvicidal effects of nanoemulsion of eucalyptus oil were reported against *A. stephensi*; a 98% mortality rate was obtained at 250 ppm [51]. However, we could not find any report on nanogel containing EGEO. The efficacy of EGEO-nanogel was more potent than the EGEO-nanoemulsion agent in the current study due to better stability.

## 5. Conclusions

1,8-cineole, *α*-pinene, *trans*-pinocarveol, aromadendrene, and globulol were identified as five major components of *E. globulus* EO (EGEO). The antibacterial and larvicidal effects of nanoemulsion and nanogel containing the EO (EGEO-nanoemulsion and EGEO-nanogel) were investigated. The efficacy of EGEO-nanogel against *S. aureus* was significantly more potent than EGEO-nanoemulsion; bacterial growth after treatment with 2500 and 5000 *μ*g/mL of EGEO-nanogel was reduced by more than 80%. Besides, 100% larval mortality was observed after treatment with 100 and 150 *μ*g/mL EGEO-nanogel. The EGEO-nanogel could thus be considered for further investigations against other pathogens and mosquito larvae.

## Figures and Tables

**Table 1:** Identified ingredients (>1%) in EGEO using GC-MS analysis.

Retention time (min)	Compound	Area (%)	Retention index
9.56	*α*-pinene	15.34	932
14.02	1,8-cineole	49.53	1026
18.89	*trans*-pinocarveol	4.32	1139
20.63	Terpinen-4-ol	1.23	1177
21.32	*α*-terpineol	1.45	1188
21.20	Dihydro carveol	1.20	1193
32.07	Aromadendrene	3.95	1441
32.93	Alloaromadendrene	2.75	1465
37.65	Spathulenol	2.81	1578
37.90	Globulol	3.69	1590
40.34	*β*-eudesmol	1.65	1650
**Total**		**87.98**	

**Table 2:** Ingredients and size analyses of EGEO-nanoemulsions.

No	EGEO (% v/v)	Tween 20 (% v/v)	Droplet size (nm)	SPAN^*∗*^
1	2	2	94.3	2.39
2	2	3	5.01	1.69
3	2	4	18.7	3.55
4	2	5	135	1.29
5	2	6	176	0.97

^*∗*^droplet size distribution.

**Table 3:** Obtained IC_50_ (*μ*g/mL) and LC_50_ (*μ*g/mL) values of the nanogel and nanoemulsion against targeted bacteria and *A. stephensi* larvae.

	*S. aureus*	*P. aeruginosa*	*A. stephensi*
Nanogel	187	>5000	32
	(29–1173)^*∗*^		(19–54)

Nanoemulsion	3732	>5000	27
	(2232->5000)		(23–33)

^*∗*^lower and upper confidence limits.

## Data Availability

All data are available from the corresponding author on reasonable request.

## Abbreviations

EO: Essential oil
GC-MS: Gas chromatography–mass spectrometry
ATR-FTIR: Attenuated total reflection-Fourier transform infrared
EGEO: *Eucalyptus globulus* essential oil
EGEO-nanoemulsion: Nanoemulsion-containing EGEO
EGEO-nanogel: Nanogel-containing EGEO.
